# Modulation of cognitive and motor functions by temporal interference stimulation: a systematic review and meta-analysis of efficacy and variability

**DOI:** 10.3389/fpsyg.2026.1932755

**Published:** 2026-09-11

**Authors:** Samaneh Aziziaram, Anna Elisabeth Fromm, Inês R. Violante, Agnes Flöel, Daria Antonenko

**Affiliations:** 1 Department of Neurology, University Medicine Greifswald, Greifswald, Germany; 2 School of Biomedical Engineering & Imaging Sciences, King’s College London, London, United Kingdom

**Keywords:** meta-analysis, non-invasive brain stimulation, response variability, stimulation effect, temporal interference stimulation (TIS), transcranial electrical stimulation (tES)

## Abstract

Temporal interference stimulation (TIS) is a non-invasive brain stimulation technique proposed to focally modulate both cortical and subcortical brain regions. A growing number of human studies have examined its effects on cognitive and motor performance, but inconsistent findings across heterogeneous protocols leave its overall behavioral efficacy uncertain. We conducted the first meta-analysis of TIS in humans, quantifying both its behavioral efficacy and the variability of responses across studies. Following PRISMA 2020 guidelines, we systematically searched PubMed, Web of Science, and Embase for human *in vivo* studies applying TIS under both active and control conditions (sham or Δ*f* = 0 Hz) and assessing behavioral outcomes in cognitive and/or motor domains. A total of 15 studies, comprising 19 outcomes and 509 participants, were included. These studies targeted both subcortical and cortical regions using envelope frequencies ranging from 5 to 130 Hz. We quantified mean stimulation effects using the standardized mean difference (Hedges’ *g*) and response variability using the log coefficient of variation ratio (lnCVR), pooled with random-effects models. Active TIS improved behavioral performance relative to the control condition [*g* = 0.30, 95% CI (0.16, 0.44), *p* < 0.001], with no detectable between-study heterogeneity (*I*^2^ = 0.0%) and consistent benefits across motor and cognitive domains. Across all outcomes, response variability was lower under active TIS than under the control condition, although this difference did not reach statistical significance [lnCVR = −0.11, 95% CI (−0.25, 0.02), *p* = 0.093]. Subgroup analyses showed significantly lower variability for motor outcomes [lnCVR = −0.26, 95% CI (−0.48, −0.04), *p* = 0.019] but not for cognitive outcomes [lnCVR = −0.03, 95% CI (−0.20, 0.14), *p* = 0.764]. Behavioral efficacy and response variability were uncorrelated across outcomes. These findings provide the first quantitative evidence that TIS improves behavioral performance across cognitive and motor domains and may reduce behavioral response variability. This combination suggests a potential benefit of TIS for producing reliable behavioral modulation, an important requirement for its further development in experimental and therapeutic neuromodulation.

## 1 Introduction

For decades, non-invasive brain stimulation (NIBS) techniques such as transcranial magnetic stimulation (TMS) and transcranial electrical stimulation (tES) have been used to modulate cognitive and motor functions in both healthy individuals and clinical populations (Patel et al., 2019; Lee et al., 2023; Lee et al., 2024; Wang et al., 2026). Their use, however, has largely been confined to cortical targets. The steep depth-to-focality trade-off of conventional NIBS forces higher stimulation intensities to reach deeper structures, which inevitably co-activates overlying cortex (Wagner et al., 2007; Deng et al., 2013). Subcortical regions such as the hippocampus, basal ganglia, and thalamus, which underpin many cognitive and motor functions and are central to the pathophysiology of disorders such as Alzheimer’s Disease (Jaroudi et al., 2017; Babcock et al., 2021), Parkinson’s Disease (Obeso et al., 2008; Citro et al., 2024), and stroke (Hochstenbach et al., 1998; Cheng et al., 2025), have therefore remained beyond the reach of conventional NIBS.

Temporal interference stimulation (TIS) was proposed as a non-invasive approach to address this limitation (Grossman et al., 2017). Two high-frequency alternating currents in the kilohertz range are delivered through separate scalp electrode pairs, with a small frequency difference (Δ*f*). Neuronal membranes act as low-pass filters that attenuate high-frequency inputs (Hutcheon and Yarom, 2000; Grossman et al., 2017). The carrier fields are therefore largely ineffective at directly driving neural activity. Where the two fields overlap, their superposition generates an amplitude-modulated envelope at the difference frequency Δ*f*, which falls within the physiological range of neural activity and can modulate neurons at the site of interference (Grossman et al., 2017; Rampersad et al., 2019). By adjusting the electrode montage and current ratio, this interference focus can be steered toward a chosen deep target (Grossman et al., 2017). Selective non-invasive modulation of deep brain structures could represent a significant advance for both fundamental neuroscience and clinical neuromodulation (Vassiliadis et al., 2026).

Human studies have provided preliminary evidence that TIS may influence behavioral performance across cognitive and motor domains in healthy young adults, older adults, and clinical populations (Violante et al., 2023; Wessel et al., 2023; Yang et al., 2025). Studies specifically assessing the safety and tolerability of TIS have generally reported only mild side effects and no serious adverse events (Piao et al., 2022; Vassiliadis et al., 2024). However, the behavioral evidence remains difficult to interpret because studies vary widely in target region, envelope frequency, stimulation intensity, control condition, and participant population, and their findings have been inconsistent. Some studies report small or null effects on cognitive and motor performance (Thiele et al., 2024; Sun et al., 2025), whereas others show notable improvements (Ma et al., 2021; Yang et al., 2025; Zhao et al., 2025).

This evidence base has so far been summarized only in qualitative reviews (Ivanov et al., 2025; Mansourinezhad et al., 2025). Demchenko et al. (2025) comprehensively reviewed 48 records (20 published studies and protocols, 28 ongoing clinical trials) of human TIS applications identified up to December 2024, spanning safety, clinical, behavioral, neuroimaging, and neurophysiological outcomes. The authors concluded that TIS is safe, well-tolerated, and capable of engaging deep brain targets in humans, with neuroimaging supporting target engagement of the primary motor cortex, basal ganglia, and hippocampus. Preliminary clinical findings further indicated acute improvements in bradykinesia and tremor in Parkinson’s Disease and essential tremor, whereas behavioral effects were variable across studies in the motor and cognitive domains. To our knowledge, no meta-analysis has yet quantified the overall behavioral efficacy of TIS in humans in controlled studies or examined response variability as a complementary outcome dimension.

Beyond mean efficacy, response variability is an important dimension of stimulation effects (López-Alonso et al., 2014; Homan et al., 2021). Stimulation can produce uniform or variable responses across individuals (López-Alonso et al., 2014). Response variability can obscure group-level effects, potentially leading to the incorrect assumption that an intervention lacks efficacy when individual differences may drive mixed outcomes (Antal et al., 2015; Nitsche et al., 2015; Fromm et al., 2025). Comparing response variability between active and control conditions provides a way to assess whether TIS alters the consistency of behavioral outcomes. Specifically, reduced variability in the active relative to the control condition would suggest that TIS produces more consistent effects across individuals, whereas increased variability would indicate that TIS amplifies differences between individuals. Quantifying such variability is therefore essential for evaluating treatment effects and for guiding the development of personalized stimulation (Homan et al., 2021; Fromm et al., 2025).

To address these gaps, the present systematic review and meta-analysis had two objectives. First, we aimed to quantify the pooled effect of TIS on behavioral performance relative to the control condition using Hedges’ g. Second, we aimed to determine whether active TIS changes the consistency of behavioral responses relative to the control condition by comparing response variability using the log coefficient of variation ratio (lnCVR). For both objectives, we examined these estimates separately within the motor and cognitive domains. To our knowledge, this is the first meta-analysis to jointly characterize the mean efficacy and response variability of TIS in humans.

## 2 Methods

### 2.1 Search strategy and study selection

**Figure 1:**
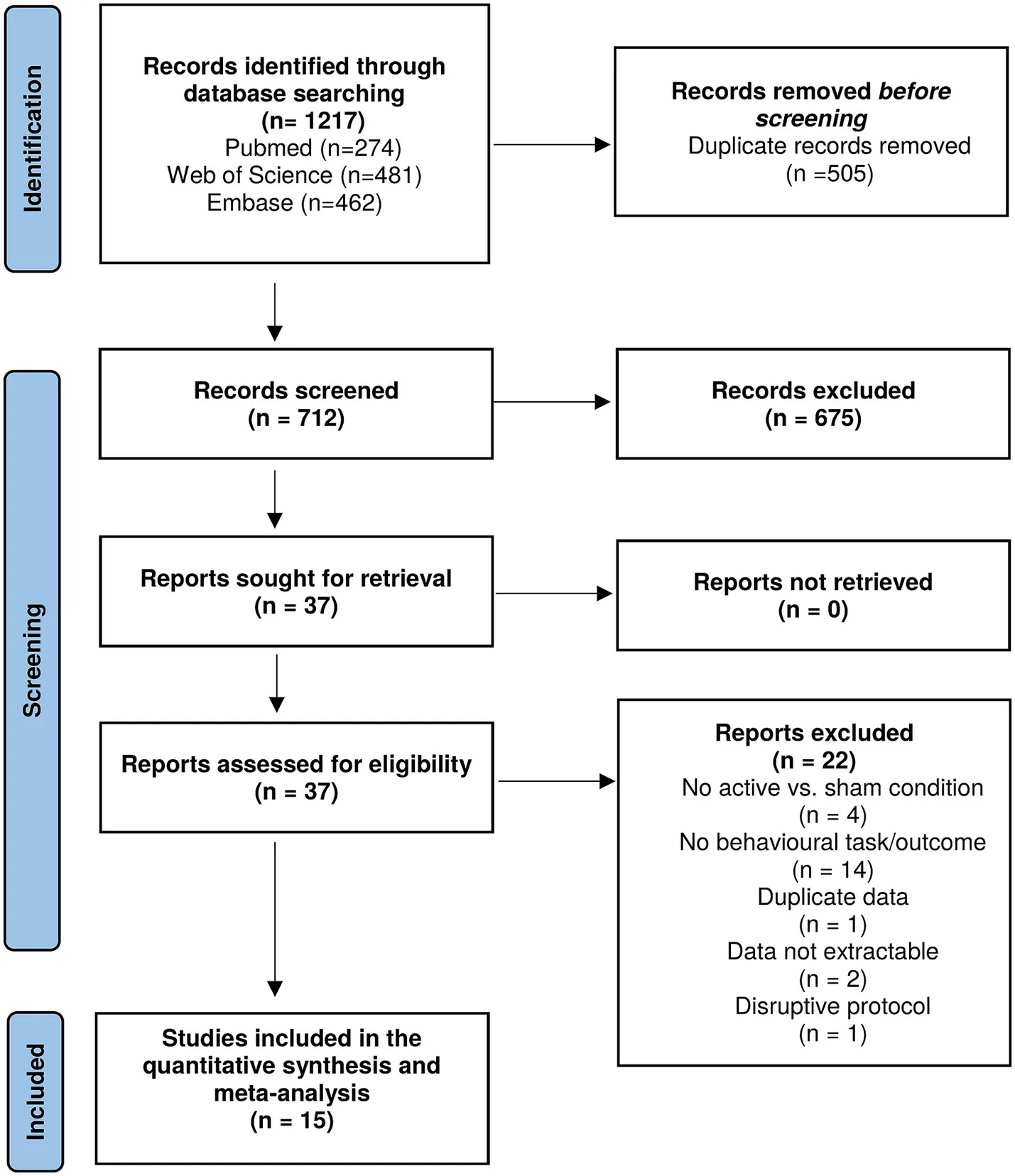
PRISMA flowchart for the study identification procedure.

This systematic review and meta-analysis was performed in accordance with the PRISMA 2020 guidelines (Page et al., 2021). A systematic literature search was conducted in PubMed, Web of Science, and Embase from inception through September 9, 2025, and subsequently updated on March 24, 2026. The search string used was: (“*temporal interference*” OR “*TI stimulation*”), with no filters applied. The reference lists of retrieved articles and relevant reviews were also screened for additional eligible studies. In total, 1,217 records were identified through database searching, comprising 1,008 from the initial search and 209 from the updated search. No additional records were identified through reference list screening (Figure 1).

Studies were included if they: (a) applied TIS *in vivo* to the human brain; (b) included both an active stimulation and a control condition (sham or high-frequency stimulation with zero envelope frequency, Δ*f* = 0 Hz); (c) assessed cognitive and/or motor functions through behavioral outcomes; (d) employed single- or multi-session protocols; (e) employed cross-over or between-subject designs; (f) were published after 2017, corresponding to the inception of TIS (Grossman et al., 2017); (g) were published in peer-reviewed journals; (h) were written in English; (i) were not case reports, case series, reviews, conference abstracts, or opinion pieces; and (j) were not designed to disrupt behavioral performance.

Motor outcomes were defined as behavioral measures of motor performance (e.g., movement accuracy and speed), motor learning and clinical motor function. Cognitive outcomes were defined as behavioral measures of performance in cognitive domains (e.g., learning and memory, executive function, and visuospatial processing). In both domains, an outcome was eligible if it was a quantitative score obtained from a task or a standardized assessment under both an active and a control condition. This systematic review was not pre-registered.

### 2.2 Data extraction

Two independent researchers (S. A. and A. E. F.) conducted the literature search, study eligibility assessment, and data extraction. Discrepancies were resolved through discussion or consultation with a third reviewer (D. A.). For each eligible study, we extracted the sample size, mean, and standard deviation (SD) of behavioral outcomes for both active and control conditions at the reported assessment timepoints (during or post-stimulation). We also extracted information on study design, participants’ characteristics, stimulation parameters, and target brain regions.

For studies in which raw summary statistics were not explicitly reported in the text, values were estimated from published figures using the *metaDigitise* R package (v1.0.1) (Pick et al., 2019). Additionally, the authors of four studies were contacted to request missing data; one responded. When raw data were not reported, alternative statistics were used. Specifically, for one study, the reported Cohen’s *d* was extracted (Tang et al., 2026), and for another, the reported *t*-statistic was used to derive the effect size (Ma et al., 2021), with both converted to Hedges’ *g* using the standard small-sample correction. In studies with multiple intervention arms, only those relevant to the research question were included. For cross-over designs with more than one active stimulation condition, a single relevant condition was selected. When studies reported more than one independent experiment, each experiment was included as a separate outcome in the meta-analysis.

To assess potential sources of bias, we conducted a narrative appraisal across four domains: allocation and blinding, outcome measurement, attrition, and reporting transparency. A standardized risk of bias tool was not applied, given the small number of included studies, their methodological heterogeneity, and the infrequent reporting of trial registration, allocation concealment, and pre-specified analysis plans.

### 2.3 Statistical analysis

To evaluate the effect of TIS on mean behavioral performance relative to the control condition, we calculated the standardized mean difference (SMD) using Hedges’ *g*, which corrects for small-sample bias and allows comparison of effects across studies using different outcome scales (Hedges, 1981; Borenstein et al., 2021). Positive SMD values indicate improved performance under TIS relative to control, whereas negative values indicate poorer performance under stimulation.

To compare the behavioral response variability between the TIS and control conditions, we calculated the log coefficient of variation ratio (lnCVR), which adjusts for mean differences between groups (Nakagawa et al., 2015; Senior et al., 2020; McCutcheon et al., 2021), using the bias-corrected formula shown below (Nakagawa et al., 2015). Positive lnCVR values indicate greater response variability under TIS than under the control condition, whereas negative values indicate lower response variability under stimulation.


$$\text{lnCVR}=\ln\frac{\frac{s_{\mathrm{TIS}}}{\bar{m_{\mathrm{TIS}}}}}{\frac{s_{\text{Control}}}{\bar{m_{\text{Control}}}}}+\frac{1}{2\;(n_{\mathrm{TIS}}-1)}-\frac{1}{2\;(n_{\text{Control}}-1)}$$

*Note*. lnCVR = log coefficient of variation ratio; *s* = standard deviation; *m* = mean; *n* = sample size.

For both SMD and lnCVR, we estimated pooled effect sizes across motor and cognitive domains using random-effects models (Borenstein et al., 2021), with a restricted maximum likelihood (REML) estimator. Separate exploratory analyses for motor and cognitive outcomes were further conducted. Finally, we conducted a Pearson correlation analysis between SMD and lnCVR to explore potential associations between stimulation effects and response variability.

Between-study heterogeneity was quantified using the estimated between-study variance (*τ*^2^) and the *I*^2^ statistic, with approximate thresholds of 25, 50, and 75% representing low, moderate, and high heterogeneity, respectively (Higgins and Thompson, 2002; Higgins et al., 2003). Additionally, Cochran’s *Q* was calculated to formally test homogeneity using a chi-square distribution. Given the limited statistical power of the *Q* test in meta-analyses with a small number of studies (Borenstein et al., 2021), statistical significance was set at *p* < 0.10.

Potential publication bias was assessed visually using funnel plots and formally using Egger’s regression test (Egger et al., 1997). The trim-and-fill procedure was also applied to estimate the number of potentially missing studies and assess the robustness of the pooled estimates (Duval and Tweedie, 2000). Additionally, leave-one-out sensitivity analyses were conducted to assess the robustness of the pooled estimates (Viechtbauer and Cheung, 2010).

All analyses were conducted in R (4.4.1, https://www.R-project.org/) using the *metafor* package to compute pooled estimates for lnCVR and SMD (Viechtbauer, 2010; Senior et al., 2020). All effects are reported with 95% confidence intervals (CIs).

## 3 Results

### 3.1 Descriptive statistics

Fifteen studies, comprising 19 outcomes, met the eligibility criteria and were included in the meta-analysis (Table 1). The studies comprised 509 participants, including healthy young adults (*n* = 433, 85%), healthy older adults (*n* = 15, 3%), individuals with methamphetamine use disorder (*n* = 38, 7.5%), and patients with Parkinson’s Disease (*n* = 23, 4.5%).

**Table 1:** Characteristics of included studies.

Study (year)	Population	Age (mean ± SD)	Sex (F/M)	Design and domain	TIS parameters	Target region	Control type	Outcomes	Task	Assessment timepoint	*g*	lnCVR
Ma et al. (2021)a	21 healthy young adults	22.43 ± 2.25	6/15	Crossover/motor	Sin/2 & 2.07 kHz/70 Hz/1 mA/30 min	Left primary motor cortex (M1)	Sham	Reaction time (ms)	Random reaction time task (RRTT)	During	0.89	—
Ma et al. (2021)b	29 healthy young adults	22.10 ± 2.02	15/14	Crossover/motor	Sin/2 & 2.02 kHz/20 Hz/1 mA/30 min	Left primary motor cortex (M1)	Sham	First Implicit Learning (ms)	Serial Reaction Time Task (SRTT)	During	0.67	—
Violante et al. (2023)a	21 healthy young adults	22.8 ± 3.2	10/11	Crossover/cognitive	Sin/2 & 2.005 kHz/5 Hz/(1 & 3 mA; 1:3)/44.5 min	Left hippocampus	Sham	Accuracy (%)	Face-name task	During	0.21	−0.09
Violante et al. (2023)b	20 healthy young adults	27.1 ± 7.6	11/9	Crossover/cognitive	Sin/2 & 2.005 kHz/5 Hz/2 mA/96 s	Left hippocampus	Sham	Accuracy (%)	Face-name task	Post	0.09	−0.02
Wessel et al. (2023)a	14 healthy young adults	23.46 ± 3.66	9/5	Crossover/motor	Theta-burst patterned/2 & 2.10 kHz/100 Hz/2 mA/30 min	Bilateral striatum	HF	Correct key presses (#)	Sequential Finger Tapping Task (SFTT)	During	0.00	−0.31
Wessel et al. (2023)b	15 healthy young adults	26.7 ± 4.27	8/7	Crossover/motor	Theta-burst patterned/2 & 2.10 kHz/100 Hz/2 mA/10.5 min	Bilateral striatum	HF	Correct sequences (#)	Sequential Finger Tapping Task (SFTT)	During	0.04	−0.19
Wessel et al. (2023)c	15 healthy older adults	66.0 ± 4.61	9/6	Crossover/motor	Theta-burst patterned/2 & 2.10 kHz/100 Hz/2 mA/10.5 min	Bilateral striatum	HF	correct sequences (#)	Sequential Finger Tapping Task (SFTT)	During	−0.04	−0.23
Beanato et al. (2024)	30 healthy young adults	23.63 ± 4.07	16/14	Crossover/Cognitive	Intermittent theta-burst patterned /2 & 2.1 kHz/100 Hz/2 mA/2 × 9 min	Right hippocampal–entorhinal complex (HC-EC)	Sham + HF	Departure time (s)	Virtual spatial navigation task	During	0.16	0.02
Thiele et al. (2024)	30 healthy young adults	TIS: 21.8 ± 2.46	TIS: 10/6	Parallel/cognitive	Sin/1 & (1 + IAF) kHz/IAF/1 mA/20 min (10 rest + 10 task)	Parieto-occipital cortex	Sham	Accuracy (%)	Shepard’s Mental Rotation Task	Post	0.23	0.08
		Sham: 22.9 ± 2.85	Sham: 11/3									
Wang et al. (2024)	59 healthy young adults	TIS: 22.97 ± 2.04	70 Hz: 15/15 Sham: 13/16	Parallel/motor	Sin/20 & 20.07 kHz/70 Hz/15 mA/30 min (50% duty)	Left primary motor cortex (M1)	Sham	Reaction time (ms)	Simple reaction time (SRT)	Post	0.01	−0.19
		Sham: 22.59 ± 3.05										
Zheng et al. (2024)	40 healthy young adults	TIS: 21.75 ± 1.80	TIS: 0/20	Parallel/motor	Sin/2 & 2.02 kHz/20 Hz/1 mA/2 × 20 min (5 days)	Primary motor cortex (M1; leg area)	Sham	Jump height (m)	Countermovement jump (CMJ)	Post	0.34	−0.61
		Sham: 21.80 ± 2.04	Sham: 0/20									
Sun et al. (2025)	20 healthy young adults	20.3 ± 2.54	14/6	Crossover/cognitive	Sin/NA/6 Hz/2 mA/20 min	Right frontoparietal network: right middle frontal gyrus and right inferior parietal lobule	Sham	Accuracy (0–1 scale)	Delayed recognition task (high-load)	Post	−0.02	0.04
Wang et al. (2025)	38 patients with methamphetamine use disorder	TIS: 36.33 ± 9.49	TIS: 0/18	Parallel/cognitive	Sin/2 & 2.01 kHz/10 Hz/1.45 & 2 mA/20 min (10 days)	Left hippocampus	Sham	Total score (#)	Montreal Cognitive Assessment (MoCA)	Post	0.63	0.06
		Sham: 33.75 ± 9.68	Sham: 0/20									
Yang et al. (2025)	12 patients with mild Parkinson’s disease	71.9 ± 4.2	7/5	Crossover/motor	Sin/1.3 & 1.43 kHz/130 Hz/2.5 and 2 mA/20 min	Right globus pallidus internus (GPi)	Sham	Total score (#)	MDS-UPDRS-III motor assessment	Post	1.38	−0.13
Zhao et al. (2025)	42 healthy young adults	NA	NA	Parallel/cognitive	Sin/2 & 2.040 kHz/40 Hz/0.75 mA/20 min	Right entorhinal cortex (EC)	Sham	Relative distance deviation (m)	Sensory-driven path integration task	Post	0.74	−0.12
Zheng et al. (2025)	25 healthy young adults	22.48 ± 1.36	15/10	Crossover/cognitive	Sin/2 & 2.006 kHz/6 Hz/1 mA/20 min	Frontoparietal network: right middle frontal gyrus and right inferior parietal lobule	Sham	d prime	N-back task (3-back)	Post	0.44	−0.12
Chen et al. (2026)	44 healthy young adults	21.70 ± 2.87	26/18	Crossover/cognitive	Sin/2 & 2.006 kHz/6 Hz/1 mA/30 min	Dorsal anterior cingulate cortex	Sham	Conflict effect (ms)	Color-word stroop task	Post	0.25	—
Li et al. (2026)	11 patients with early- to mid-stage Parkinson’s disease	71.91 ± 4.44	6/5	Crossover /motor	Sin/1.30 & 1.43 kHz/130 Hz/2.5 & 2 mA /20 min	Right globus pallidus internus (GPi)	Sham	Stride length variability (%)	Timed Up and Go (TUG) test (dual-task condition)	Post	−0.08	0.18
Tang et al. (2026)	23 healthy young adults	20.31 ± 1.69	0/23	Crossover/motor	Sin/2 & 2.02 kHz/20 Hz/5 mA/20 min	Right striatum	Sham	First Implicit Learning (ms)	Serial Reaction Time Task (SRTT)	Post	0.10	—

TIS parameters are reported as: waveform type (sinusoidal or patterned)/carrier frequencies (kHz)/envelope frequency (Hz)/intensity (mA, zero-to-peak)/duration (min). Letters a, b, and c denote independent experiments from the same publication. F, female; *g*, Hedges’ *g*; GPi, globus pallidus internus; HC–EC, hippocampal–entorhinal complex; HF, high frequency; IAF, individual alpha frequency; lnCVR, log coefficient of variation ratio; M, male; MDS-UPDRS-III, Movement Disorder Society–Unified Parkinson’s Disease Rating Scale part III; NA, not available; SD, standard deviation; TIS, transcranial temporal interference stimulation.

Seven studies (10 outcomes) investigated motor functions (Ma et al., 2021; Wessel et al., 2023; Wang et al., 2024; Zheng et al., 2024; Yang et al., 2025; Li et al., 2026; Tang et al., 2026) and eight (9 outcomes) examined cognitive functions (Violante et al., 2023; Beanato et al., 2024; Thiele et al., 2024; Sun et al., 2025; Wang et al., 2025; Zhao et al., 2025; Zheng et al., 2025; Chen et al., 2026). Motor outcomes included explosive power assessed via the countermovement jump (Zheng et al., 2024); motor and visuomotor response speed measured using simple reaction time (Wang et al., 2024) and random reaction time tasks (Ma et al., 2021); clinical motor function in patient populations evaluated using the MDS-UPDRS-III (Yang et al., 2025) and the Timed Up and Go test (Li et al., 2026); and motor learning and sequence acquisition assessed using the Serial Reaction Time Task (Ma et al., 2021; Tang et al., 2026) and Sequential Finger Tapping Task (Wessel et al., 2023). Cognitive outcomes included working memory assessed using the N-back task (Zheng et al., 2025) and delayed recognition tasks (Sun et al., 2025); episodic memory via face-name association (Violante et al., 2023); visuospatial processing assessed via mental rotation tasks (Thiele et al., 2024); spatial navigation and path integration measured using virtual navigation (Beanato et al., 2024) and sensory-driven path integration tasks (Zhao et al., 2025); executive control examined with the color-word Stroop task (Chen et al., 2026); and global cognitive status evaluated using the Montreal Cognitive Assessment (Wang et al., 2025).

Thirteen studies employed a single-session stimulation protocol, whereas two studies used a multi-session protocol (Zheng et al., 2024; Wang et al., 2025). Five studies used a between-subjects design (Thiele et al., 2024; Wang et al., 2024, 2025; Zheng et al., 2024; Zhao et al., 2025), while 10 adopted a cross-over design. Behavioral outcomes were assessed during stimulation in four studies (Ma et al., 2021; Violante et al., 2023; Wessel et al., 2023; Beanato et al., 2024) and post-stimulation in the remaining 11. Eight studies targeted deep brain regions (Violante et al., 2023; Wessel et al., 2023; Beanato et al., 2024; Wang et al., 2025; Yang et al., 2025; Zhao et al., 2025; Li et al., 2026; Tang et al., 2026), whereas seven targeted superficial cortical regions (Ma et al., 2021; Thiele et al., 2024; Wang et al., 2024; Zheng et al., 2024; Sun et al., 2025; Zheng et al., 2025; Chen et al., 2026).

The SMD analysis included all 19 outcomes (10 motor, 9 cognitive). For the lnCVR analysis, 15 outcomes were included (7 motor, 8 cognitive), with four outcomes excluded because separate within-condition means and SDs were either unavailable or not appropriate for estimating condition-specific response variability. The Pearson correlation between SMD and lnCVR was conducted on the 15 outcomes for which both effect-size estimates were available.

The narrative appraisal indicated that allocation and blinding were sufficiently reported in most studies. As required by the inclusion criteria, all included studies compared active TIS with a control condition, either sham or high-frequency stimulation with a zero-envelope frequency. Allocation of group or of condition order was randomized in most studies, while several cross-over studies relied on counterbalanced or Latin square ordering. This approach balances period effects but does not constitute random sequence generation. Most studies reported blinding of participants, and about half formally assessed blinding efficacy. Outcome measurement was consistent across studies, with most relying on computerized or instrumented tasks and a minority on examiner-administered clinical rating scales. Attrition was well documented. Enrolled and analyzed sample sizes were reported in most studies, and exclusions were generally based on data-quality or performance criteria. Allocation concealment, trial registration, and pre-specified analysis plans were described only infrequently.

### 3.2 Stimulation effects on mean performance

**Figure 2:**
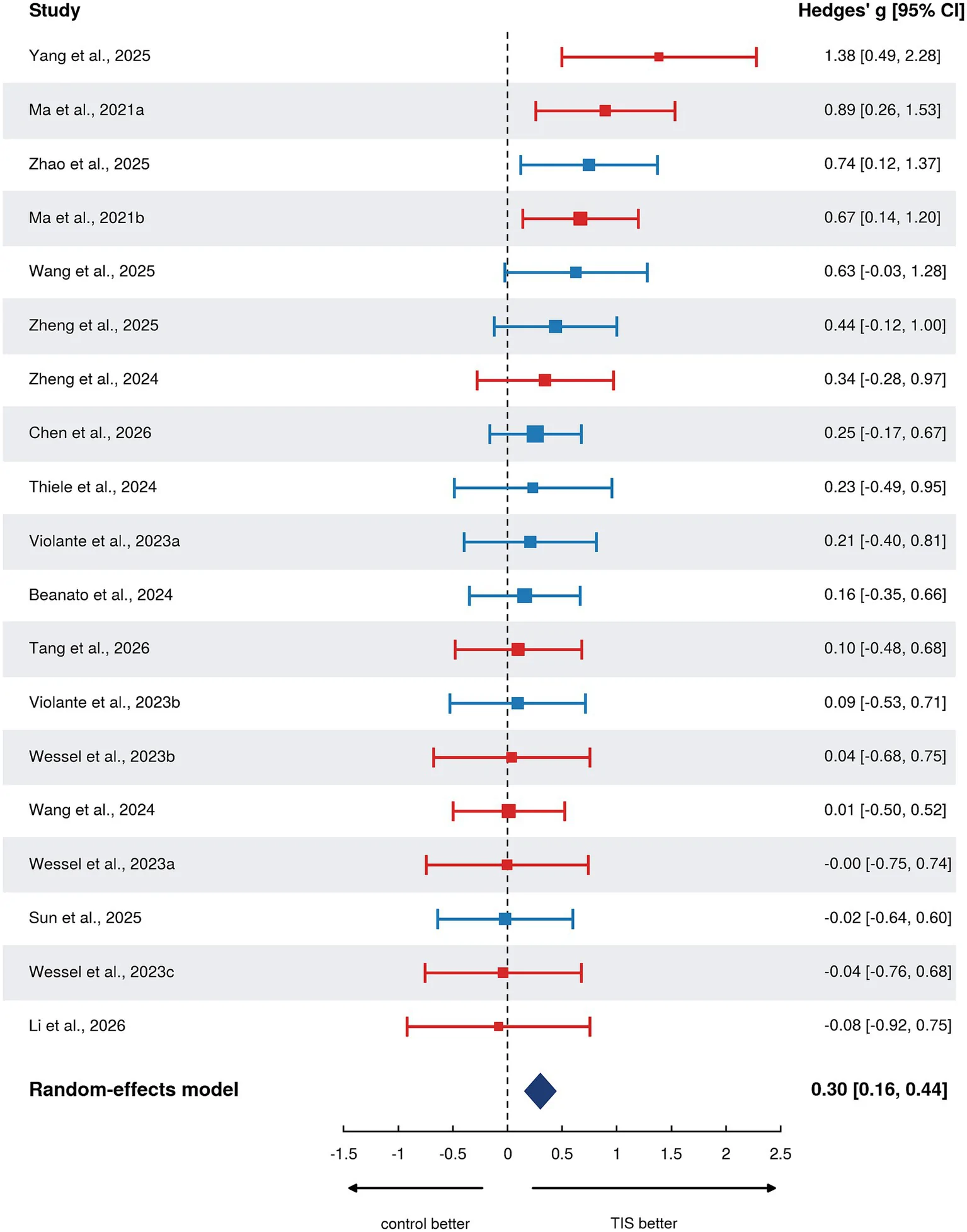
Stimulation effects on mean performance. Forest plot of standardized mean differences (SMD; Hedges’ *g*) comparing temporal interference stimulation (TIS) with the control condition across 19 outcomes. Positive values indicate improved performance under TIS relative to control. Squares represent outcome-level effect size; horizontal lines represent 95% confidence intervals; colors denote task domain (blue = cognitive, red = motor). The diamond represents the pooled random-effects estimate. Outcomes are ordered by the magnitude of the SMD. CI, confidence interval; SMD, standardized mean difference.

The random-effects meta-analysis revealed better overall mean performance under TIS compared with the control condition [*g* = 0.30, 95% CI (0.16, 0.44), *p* < 0.001]. Between-study heterogeneity was negligible (*τ*^2^ = 0.00, *I*^2^ = 0.0%, *Q* = 20.6, *p* = 0.301; Figure 2).

**Figure 3:**
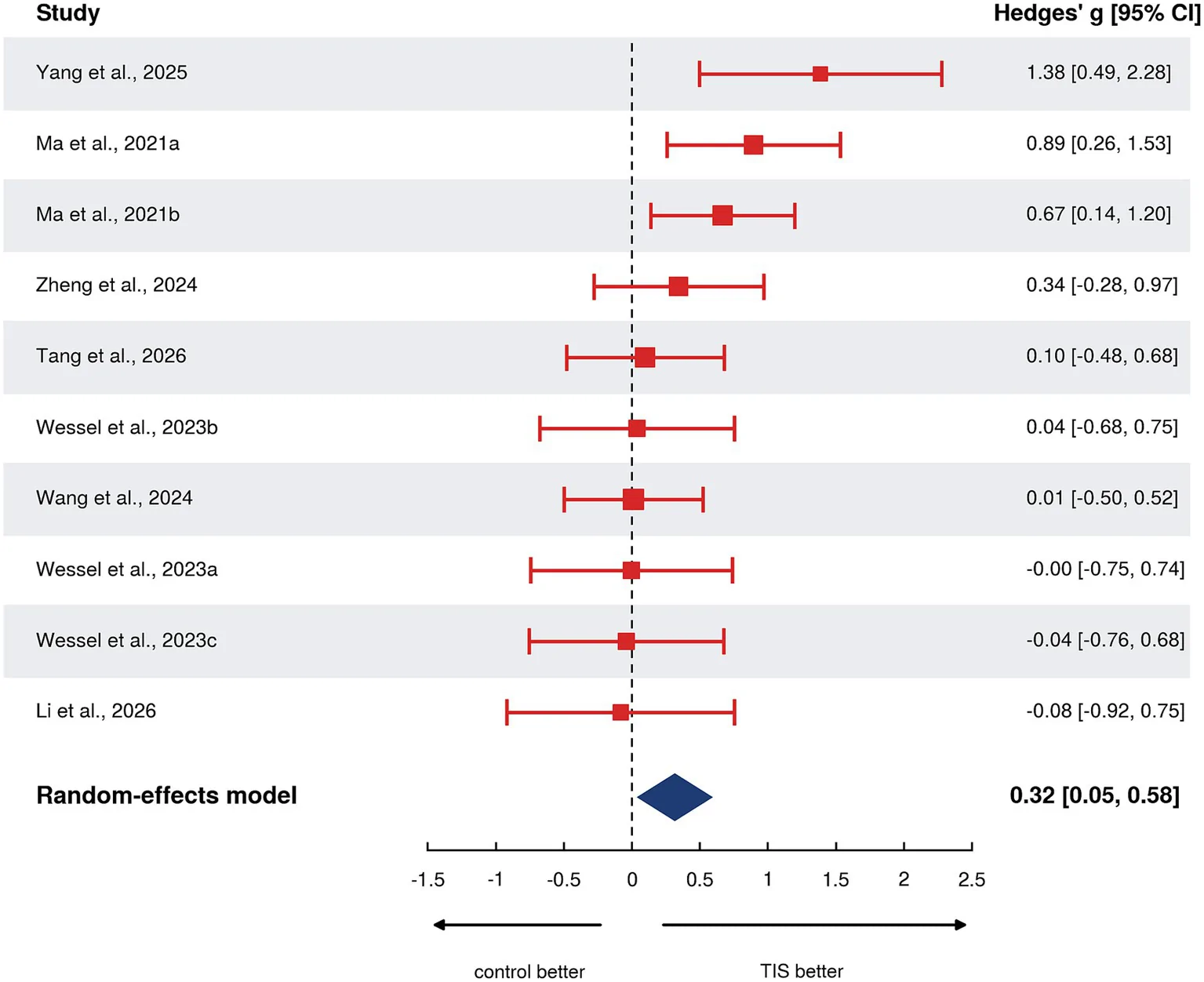
Stimulation effects on mean performance for motor outcomes. Forest plot of standardized mean differences (SMD; Hedges’ *g*) comparing temporal interference stimulation (TIS) with the control condition for 10 motor outcomes. Positive values indicate improved performance under TIS relative to control. Squares represent outcome-level effect sizes; horizontal lines indicate 95% confidence intervals. The diamond represents the pooled random-effects estimate. Outcomes are ordered by the magnitude of the SMD. CI, confidence interval; SMD, standardized mean difference.

**Figure 4:**
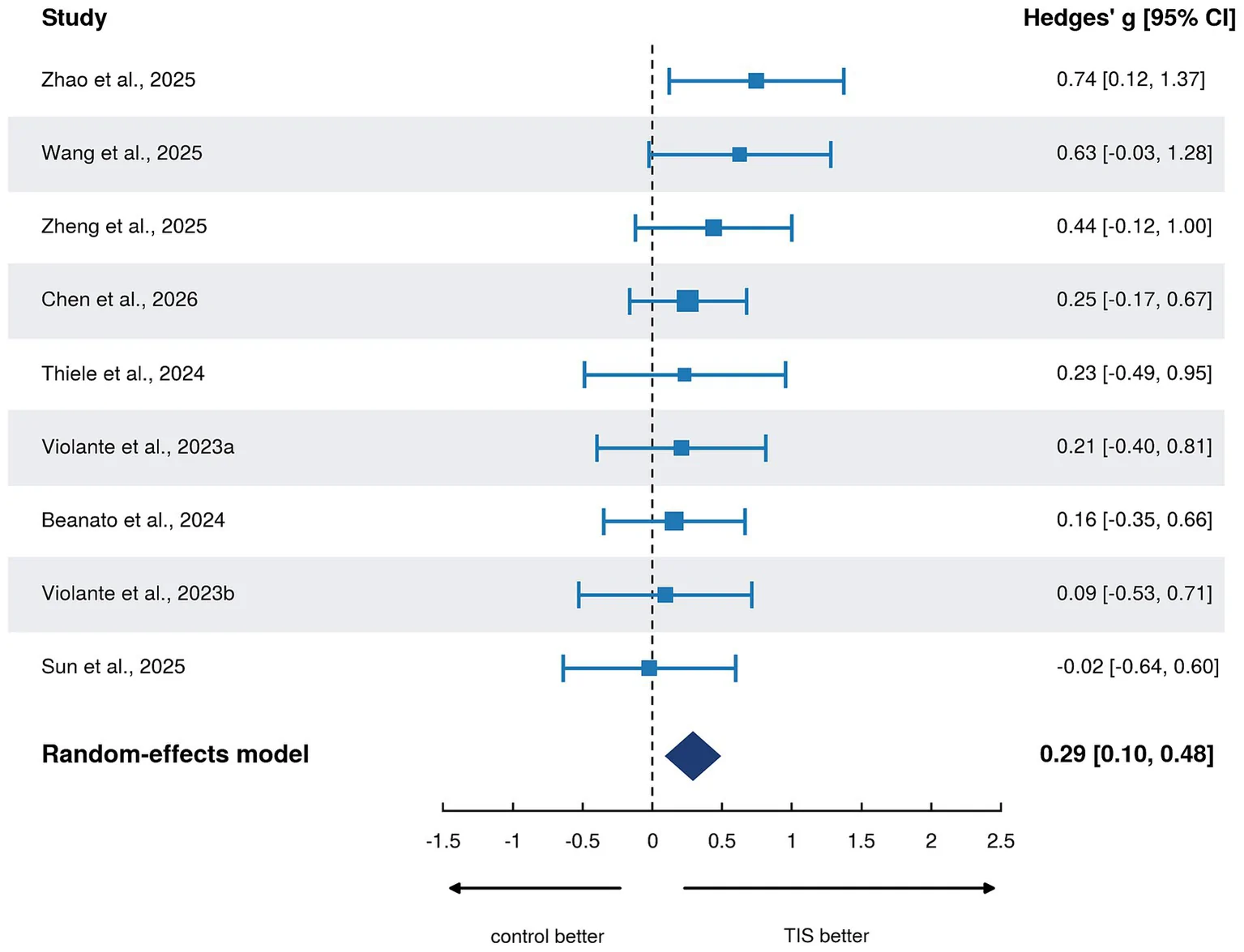
Stimulation effects on mean performance for cognitive outcomes. Forest plot of standardized mean differences (SMD; Hedges’ *g*) comparing temporal interference stimulation (TIS) with the control condition for 9 cognitive outcomes. Positive values indicate improved performance under TIS relative to control. Squares represent outcome-level effect sizes; horizontal lines indicate 95% confidence intervals. The diamond represents the pooled random-effects estimate. Outcomes are ordered by the magnitude of the SMD. CI, confidence interval; SMD, standardized mean difference.

Exploratory analyses revealed improved performance in both motor [*g* = 0.32, 95% CI (0.05, 0.58), *p* = 0.020; Figure 3] and cognitive domains [*g* = 0.29, 95% CI (0.10, 0.48), *p* = 0.003; Figure 4] under TIS relative to the control condition. Between-study heterogeneity was moderate for motor outcomes (*τ*^2^ = 0.07, *I*^2^ = 38.6%, *Q* = 15.5, *p* = 0.079) and negligible for cognitive outcomes (*τ*^2^ = 0.00, *I*^2^ = 0.0%, *Q* = 5.1, *p* = 0.748).

There was little evidence of publication bias. Egger’s regression test suggested approximate funnel plot symmetry [*t* = 0.55, *p* = 0.590; limit estimate: *b* = 0.06, 95% CI (−0.87, 0.99); Supplementary Figure S1]. Trim-and-fill analysis did not identify any missing studies (*k*_0_ = 0) and the pooled estimate remained unchanged after adjustment [*g* = 0.30, 95% CI (0.16, 0.44)].

Leave-one-out sensitivity analysis supported the robustness of the overall SMD results, with pooled estimates ranging from 0.27 to 0.32 and 95% CIs remaining above zero across all iterations. The *I*^2^ values consistently indicated low heterogeneity (range: 0.0–8.5%; Supplementary Table S1).

### 3.3 Stimulation effects on response variability

**Figure 5:**
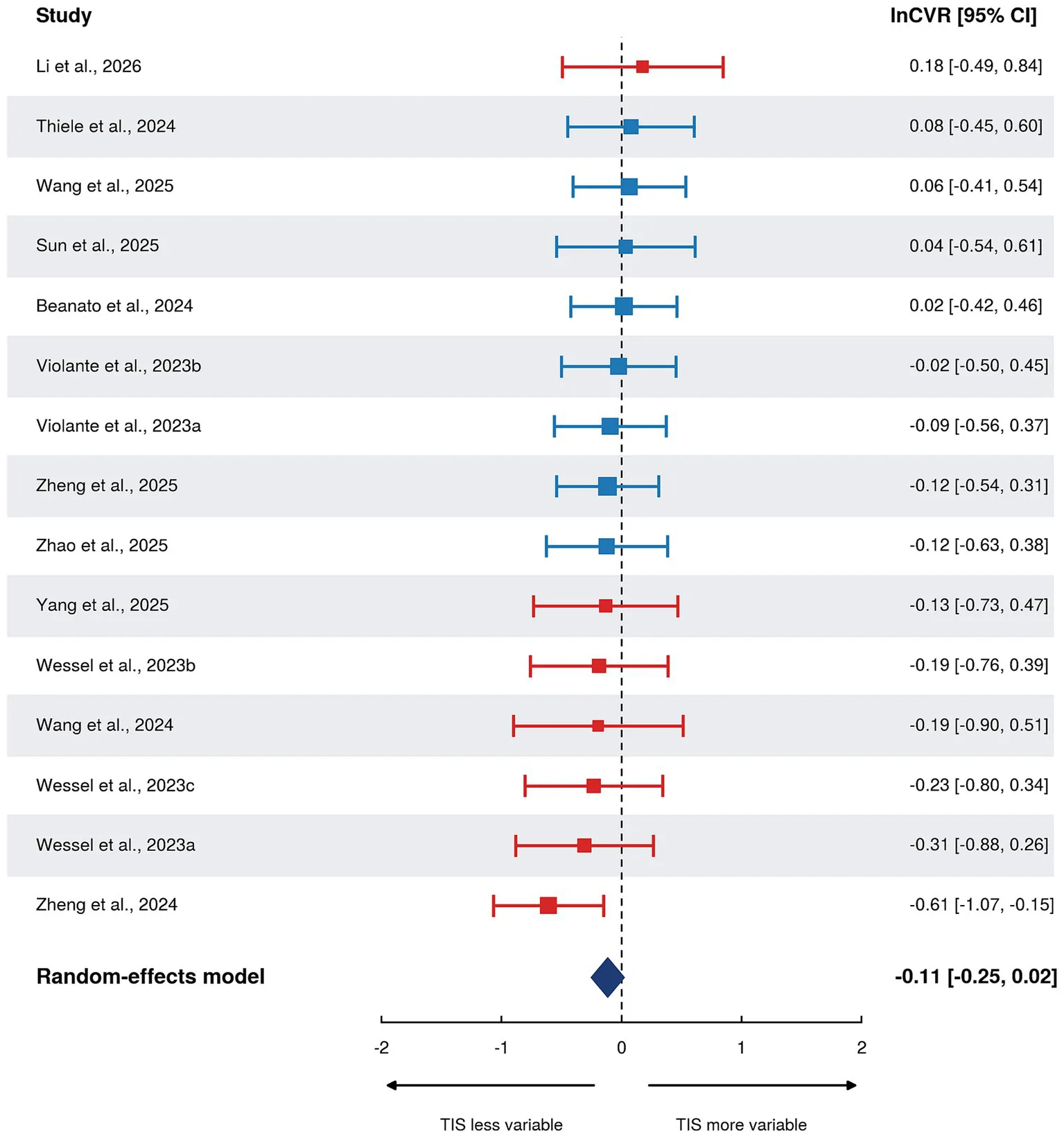
Stimulation effects on response variability. Forest plot of the log coefficient of variation ratio (lnCVR) comparing temporal interference stimulation (TIS) with the control condition across 15 outcomes. Positive lnCVR values indicate greater response variability under TIS compared to control. Squares represent outcome-level effect sizes; horizontal lines indicate 95% confidence intervals; colors denote task domain (blue = cognitive, red = motor). The diamond represents the pooled random-effects estimate. Outcomes are ordered by the magnitude of lnCVR. CI, confidence interval; lnCVR, log coefficient of variation ratio.

The random-effects meta-analysis of lnCVR revealed lower, though non-significant, response variability under TIS relative to the control condition [lnCVR = −0.11, 95% CI (−0.25, 0.02), *p* = 0.093]. Between-study heterogeneity was negligible (*τ*^2^ = 0.00, *I*^2^ = 0.0%, *Q* = 7.7, *p* = 0.903; Figure 5).

**Figure 6:**
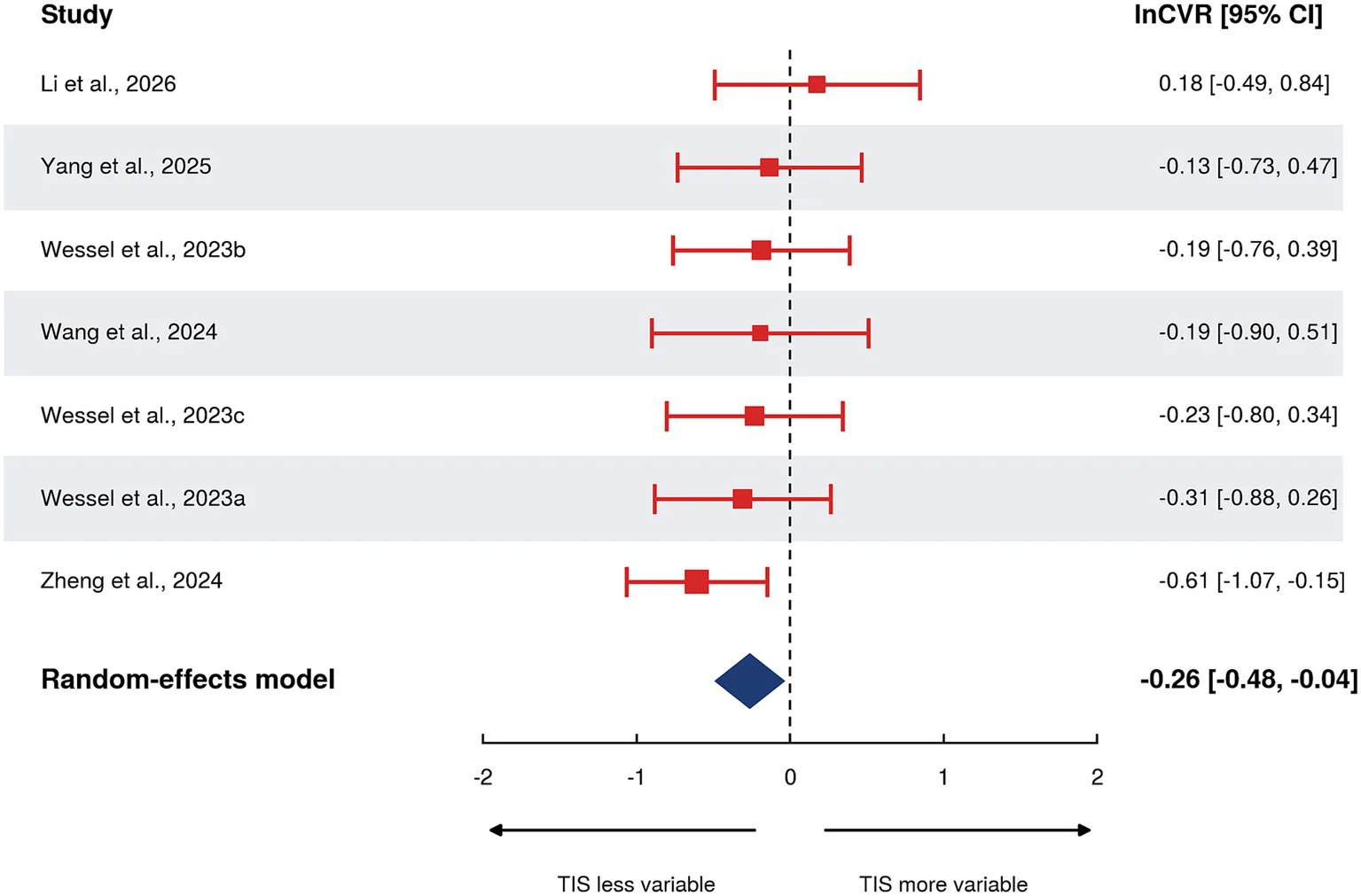
Stimulation effects on response variability for motor outcomes. Forest plot of the log coefficient of variation ratio (lnCVR) comparing temporal interference stimulation (TIS) with the control condition for 7 motor outcomes. Positive lnCVR values indicate greater response variability under TIS compared to control. Squares represent outcome-level effect sizes; horizontal lines indicate 95% confidence intervals. The diamond represents the pooled random-effects estimate. Outcomes are ordered by the magnitude of lnCVR. lnCVR, log coefficient of variation ratio; CI, confidence interval.

**Figure 7:**
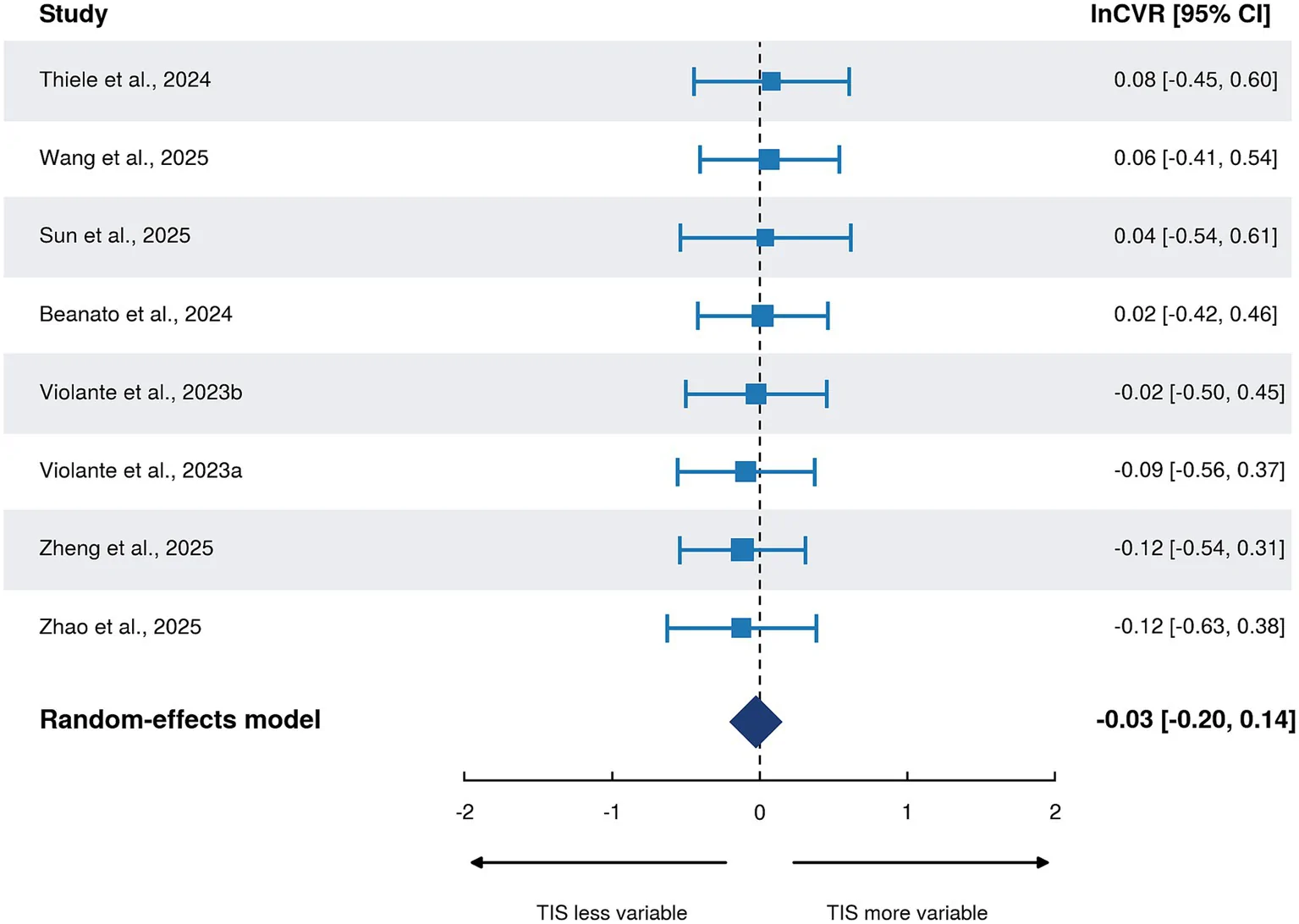
Stimulation effects on response variability for cognitive outcomes. Forest plot of the log coefficient of variation ratio (lnCVR) comparing temporal interference stimulation (TIS) with the control condition for 8 cognitive outcomes. Positive lnCVR values indicate greater response variability under TIS compared to control. Squares represent outcome-level effect sizes; horizontal lines indicate 95% confidence intervals. The diamond represents the pooled random-effects estimate. Outcomes are ordered by the magnitude of lnCVR. lnCVR, log coefficient of variation ratio; CI, confidence interval.

In the exploratory analyses, motor outcomes showed significantly lower response variability under TIS compared with the control condition [lnCVR = −0.26, 95% CI (−0.48, −0.04), *p* = 0.019], with no evidence of between-study heterogeneity [*τ*^2^ = 0.00, *I*^2^ = 0.0%, *Q* = 4.2, *p* = 0.656; Figure 6]. For cognitive outcomes, response variability was similar between conditions [lnCVR = −0.03, 95% CI (−0.20, 0.14), *p* = 0.764], with no evidence of heterogeneity (*τ*^2^ = 0.00, *I*^2^ = 0.0%, *Q* = 0.8, *p* = 0.998; Figure 7).

Egger’s regression test indicated no significant funnel plot asymmetry [*t* = 0.29, *p* = 0.774; limit estimate: *b* = −0.22, 95% CI (−1.02, 0.58)]. The trim-and-fill analysis identified two potentially missing studies on the left side of the funnel plot, suggesting an underrepresentation of smaller studies with more negative effects. After adjustment, the pooled estimate remained consistent and became stronger [lnCVR = −0.14, 95% CI (−0.27, −0.01), *p* = 0.029], indicating that any publication bias may have led to an underestimation of reduced variability under stimulation. Between-study heterogeneity remained negligible both before and after the trim-and-fill adjustment (*τ*^2^ = 0.00, *I*^2^ = 0.0%; Supplementary Figure S2).

Leave-one-out sensitivity analysis confirmed the robustness of the overall lnCVR results, with pooled estimates ranging from −0.13 to −0.07, with 95% CIs including zero across all iterations. Between-study heterogeneity remained negligible throughout the analysis (*I*^2^ = 0.0% in all iterations; Supplementary Table S2).

### 3.4 Association between stimulation effects and response variability

**Figure 8:**
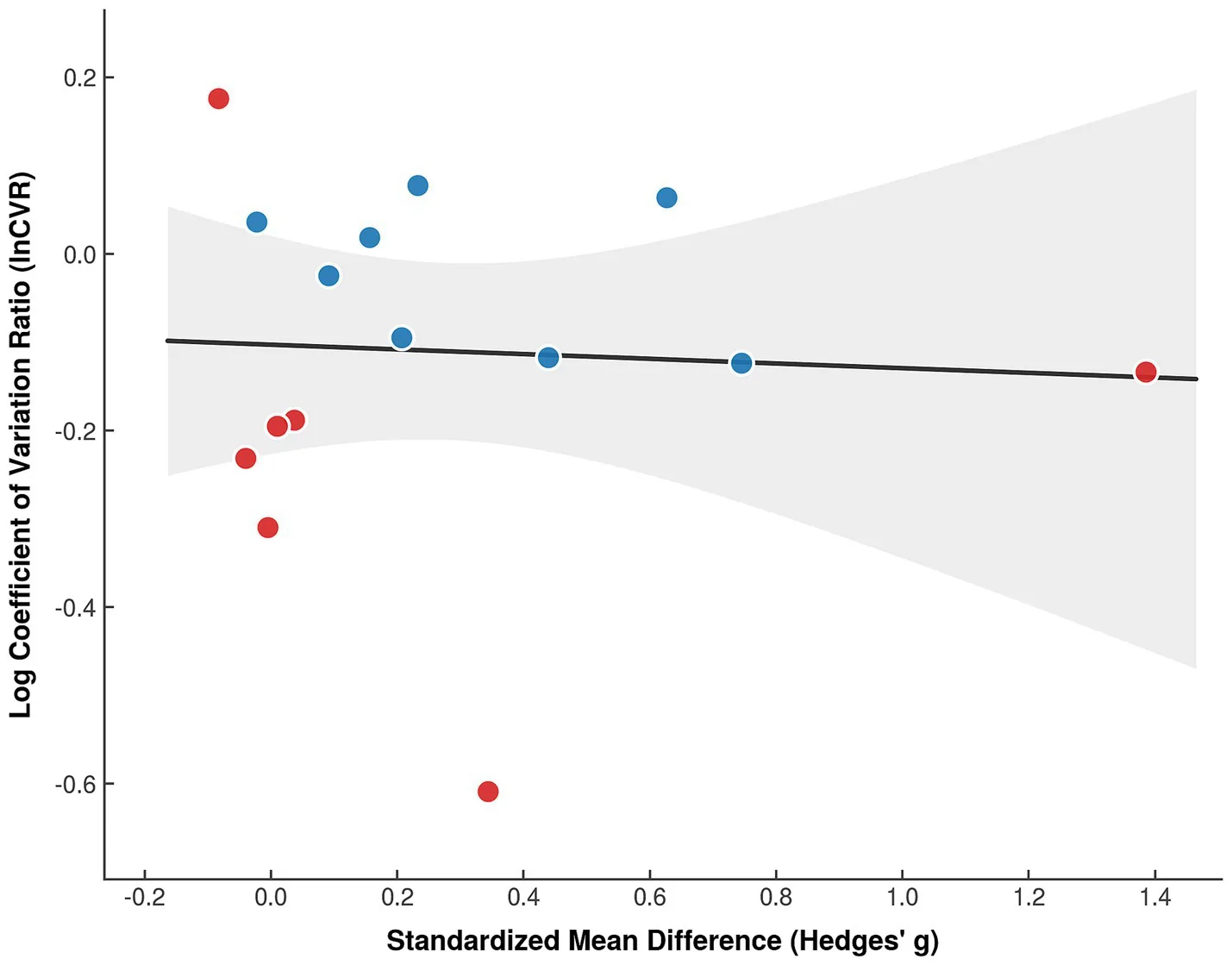
Association between standardized mean difference and response variability. Scatter plot illustrating the association between standardized mean difference (SMD; Hedges’ *g*) and the log coefficient of variation ratio (lnCVR) across 15 matched outcomes. Each point represents an individual outcome; colors indicate task domain (blue = cognitive, red = motor); the solid line represents the fitted linear regression; the shaded band indicates the 95% confidence interval.

The association between SMD and lnCVR was non-significant [*r* = −0.06, 95% CI (−0.55, 0.47), *p* = 0.846; Figure 8].

## 4 Discussion

This meta-analysis quantified the behavioral effects of TIS in humans across motor and cognitive domains. Active TIS improved behavioral performance relative to control, with a small pooled effect that was consistent across both domains and showed no detectable between-study heterogeneity. Additionally, active TIS was associated with more homogeneous responses across participants relative to control, an effect that appeared to be driven primarily by motor outcomes. Effect size and response variability were not correlated across outcomes. These findings provide the first quantitative synthesis of TIS behavioral efficacy and characterize response variability as a complementary dimension of TIS outcomes.

Regarding the behavioral efficacy of TIS, our pooled estimate showed that, across studies, the behavioral effect was positive and statistically reliable. This effect was small, and its magnitude aligns with what has been reported for conventional non-invasive stimulation. A meta-analysis of anodal tDCS over the motor cortex found a small improvement in physical performance in healthy adults (Hedges’ *g* = 0.29) (Winker et al., 2024). Comparable small effects have been documented for tDCS on cognition-related reaction time in older adults (SMD = 0.17) (Lee et al., 2021) and for tACS on cognitive performance in healthy young adults (SMD = 0.16) (Lee et al., 2023). This suggests that TIS modulates behavior to a degree comparable with conventional transcranial stimulation techniques, despite its capacity to focally engage subcortical targets. Such a modest effect is consistent with the known biophysical properties of the technique. TIS delivers a high-frequency carrier that is amplitude-modulated at the low-frequency envelope, and neurons must demodulate this signal to respond to it. This demodulation is incomplete and, together with the frequency-dependent shunting of current through scalp and skull, attenuates the neural response (Vassiliadis et al., 2026). The discrepancy with null findings in individual studies may reflect the small sample sizes that characterize the current TIS literature (Mansourinezhad et al., 2025). This factor has been identified in prior work on conventional electrical stimulation as a major source of the inconsistent and poorly replicable effects reported across individual tDCS trials (Minarik et al., 2016). Demchenko et al. (2025) similarly described the behavioral effects of TIS as variable across studies and identified small sample sizes and methodological differences as key limitations of the current evidence base.

The effect was evident in both motor and cognitive domains. Motor improvements were observed in an explosive-power task after primary motor cortex stimulation (Zheng et al., 2024), in a reaction-time task during primary motor cortex stimulation (Ma et al., 2021), and in a clinical motor assessment after globus pallidus internus stimulation (Yang et al., 2025). Cognitive improvements were observed in hippocampal stimulation during an associative memory task (Violante et al., 2023) and during repeated sessions in methamphetamine use disorder (Wang et al., 2025), in hippocampal–entorhinal stimulation during virtual spatial navigation (Beanato et al., 2024), and in frontoparietal stimulation during a high-load working-memory task (Zheng et al., 2025). The presence of positive effects across different target regions, tasks, and a population spanning healthy young adults, healthy older adults, and patients with Parkinson’s Disease and methamphetamine use disorder underscores that the pooled effect reflects a general capacity of TIS to modulate behavior (Mansourinezhad et al., 2025; Vassiliadis et al., 2026).

The individual effect sizes are also informative. The largest effect across studies was observed in patients with Parkinson’s Disease, where globus pallidus internus stimulation improved motor symptoms measured with the MDS-UPDRS-III (*g* = 1.38) (Yang et al., 2025). Consistent with this, the review by Demchenko et al. (2025) described acute motor improvements following single-session TIS of basal ganglia targets in Parkinson’s Disease, while noting that the available studies included small samples without long-term follow-up. Together, these findings are encouraging for the clinical translation of TIS. Across the included outcomes, improvements were similar for deep and superficial targets, and behavioral efficacy did not appear to depend on target depth. Effects were also similar for outcomes assessed during and after stimulation. Both multi-session protocols produced effects above the pooled estimate (Zheng et al., 2024; Wang et al., 2025). These observations were not tested formally and should be regarded as hypothesis generating.

Further, the efficacy estimate was robust. Leave-one-out analysis kept the pooled effect positive and significant, and trim-and-fill identified no missing studies, indicating little influence of publication bias on the average effect. Between-study heterogeneity was negligible despite variation in target region, envelope frequency, intensity, and population, which suggests that the direction and approximate magnitude of the behavioral effect were consistent across these methodological differences. Given the limited number of available studies, this absence of heterogeneity should be interpreted with caution, as a small evidence base reduces the power to detect it. Overall, the conclusions of this first quantitative meta-analysis are consistent with previous reviews reporting preliminary evidence of TIS efficacy (Ivanov et al., 2025; Mansourinezhad et al., 2025).

Response variability was slightly lower under active TIS in comparison to the control condition, an effect which seems to be primarily driven by motor outcomes. Previous research in brain stimulation and pharmacological treatment has introduced response variability as a marker of treatment consistency, indicating whether a group-level mean effect reflects a uniform response across individuals or a heterogeneous subgroup pattern (Homan et al., 2021; McCutcheon et al., 2021). A higher variability under active relative to the control condition could suggest a heterogeneous response, which may reflect favorable or unfavorable profiles of responsiveness across individuals, whereas lower variability would indicate a more homogeneous response to treatment across individuals (Homan et al., 2021; McCutcheon et al., 2021). Examining this dimension is therefore meaningful even when mean effects are present, as it provides insight into the generalizability and individual reliability of the intervention. Homan et al. (2021) examined treatment variability in clinical trials of TMS and tDCS across psychiatric diagnoses. Their results showed consistently low variability in treatment effects, except for schizophrenia, suggesting that the need for personalized brain stimulation in these populations remains an open question. A meta-analysis of tDCS effects on response inhibition addressed a similar question in healthy and clinical samples. The authors reported no difference in variability between active and sham conditions, suggesting that inter-individual differences may not substantially contribute to the heterogeneity in this domain (Lasogga et al., 2024).

Although the overall reduction in response variability did not reach statistical significance, response variability was significantly lower under active TIS for motor outcomes, but no comparable reduction was observed for cognitive outcomes. This domain-specific pattern may reflect the greater complexity of cognitive performance, which is influenced by a broader range of factors beyond the stimulated network, including individual differences in strategy, baseline ability, and attentional state (Hsu et al., 2016; Vergallito et al., 2022). These sources of variance may make stimulation-related changes in response consistency more difficult to detect, even when mean performance improves. This interpretation is consistent with evidence that non-invasive brain stimulation effects on cognitive outcomes may be more sensitive to moderating factors than effects on motor outcomes (Jacobson et al., 2012). However, the present analysis cannot directly determine the factors responsible for the observed domain-specific pattern, and these explanations should therefore be considered tentative.

The mean stimulation effect and response variability were not associated across outcomes. In our recent tDCS meta-analysis, lower response variability was associated with greater stimulation efficacy, particularly in older adults (Fromm et al., 2025). A comparable inverse pattern was observed in antipsychotic treatment for schizophrenia, where higher efficacy was associated with lower response variability across trials (McCutcheon et al., 2021). In contrast, the variance meta-analysis of TMS and tDCS in psychiatric populations found no relationship between mean effect and variability (Homan et al., 2021). The null association observed in the present data should therefore be interpreted with caution, particularly given the small number of contributing outcomes.

In summary, TIS produced a small but reliable behavioral benefit that did not come at the cost of greater response variability, indicating that its effects are reasonably stable across individuals rather than confined to particular responders. This is an encouraging sign for the technique’s translational potential as a non-invasive neuromodulation approach.

### 4.1 Limitations

Some limitations of our study should be considered. The number of eligible studies was small, with 15 studies contributing 19 outcomes, which constrained statistical power and precluded formal subgroup analyses of the stimulation parameters most likely to moderate TIS effects, including target region, envelope frequency, intensity, and montage (Mansourinezhad et al., 2025; Vassiliadis et al., 2026). The included samples were also dominated by healthy young adults, with patients and older adults contributing only a minority of outcomes, which limits the generalizability of the pooled estimates to the clinical populations for whom TIS is ultimately intended. Furthermore, some included studies reported normalized performance scores rather than raw behavioral data in their original reports. For these studies, raw values were entered into the meta-analysis to maintain consistency across studies. As a result, the calculated effect sizes may be conservative and may underestimate the magnitude of the behavioral effects reported in the original studies.

## 5 Conclusion

Our findings provide the first quantitative evidence that active TIS improves behavioral performance across motor and cognitive domains. Moreover, our results suggest that TIS may reduce response variability compared to a control condition, leading to more homogeneous responses, particularly in motor outcomes. Future studies with larger samples, standardized protocols, and individualized targeting approaches will be needed to clarify the factors that shape behavioral efficacy and response variability under TIS, particularly in clinical populations and multi-session interventions.

## Data Availability

The original contributions presented in the study are publicly available. This data can be found here: Open Science Framework (OSF) repository at https://osf.io/sv6cu.

## References

[ref1] Antal A. Keeser D. Priori A. Padberg F. Nitsche M. (2015). Conceptual and procedural shortcomings of the systematic review “evidence that transcranial direct current stimulation (tDCS) generates little-to-no reliable neurophysiologic effect beyond MEP amplitude modulation in healthy human subjects: a systematic review” by Horvath and co-workers. Brain Stimul. 8, 846–849. doi: 10.1016/j.brs.2015.05.010, 26143023

[ref2] Babcock K. R. Page J. S. Fallon J. R. Webb A. E. (2021). Adult hippocampal neurogenesis in aging and Alzheimer’s disease. Stem Cell Rep 16, 681–693. doi: 10.1016/j.stemcr.2021.01.019, 33636114 PMC8072031

[ref3] Beanato E. Moon H. J. Windel F. Vassiliadis P. Wessel M. J. Popa T. . (2024). Noninvasive modulation of the hippocampal-entorhinal complex during spatial navigation in humans. Sci. Adv. 10:eado4103. doi: 10.1126/sciadv.ado4103, 39475597 PMC11524170

[ref4] Borenstein M. Hedges L. V. Higgins J. P. Rothstein H. R. (2021). Introduction to Meta-Analysis. Chichester, UK: John Wiley & Sons.

[ref5] Chen X. Zeng G. Q. Ma R. Li N. Zhang M. Zhang X. (2026). The distinct roles of the dorsolateral prefrontal cortex and dorsal anterior cingulate cortex in cognitive control: evidence from transcranial temporal interference stimulation. Psychophysiology 63:e70269. doi: 10.1111/psyp.70269 41808199

[ref6] Cheng Q. Liu S. Wang J. Wang Y. Han B. Wang L. . (2025). Alterations in the functional connectivity of thalamic subregions after basal ganglia stroke. Front. Neurol. 16:1584290. doi: 10.3389/fneur.2025.1584290, 40546256 PMC12178861

[ref7] Citro S. Lazzaro G. D. Cimmino A. T. Giuffrè G. M. Marra C. Calabresi P. (2024). A multiple hits hypothesis for memory dysfunction in Parkinson disease. Nat. Rev. Neurol. 20, 50–61. doi: 10.1038/s41582-023-00905-z, 38052985

[ref8] Demchenko I. Tailor I. Chegini S. Yu H. Nezhad F. G. Rueda A. . (2025). Human applications of transcranial temporal interference stimulation: a systematic review. Brain Stimul. 18, 2054–2066. doi: 10.1016/j.brs.2025.10.023 41167554

[ref9] Deng Z.-D. Lisanby S. H. Peterchev A. V. (2013). Electric field depth–focality tradeoff in transcranial magnetic stimulation: simulation comparison of 50 coil designs. Brain Stimul. 6, 1–13. doi: 10.1016/j.brs.2012.02.005, 22483681 PMC3568257

[ref10] Duval S. Tweedie R. (2000). Trim and fill: a simple funnel-plot–based method of testing and adjusting for publication bias in meta-analysis. Biometrics 56, 455–463. doi: 10.1111/j.0006-341x.2000.00455.x 10877304

[ref9001] Egger M. Davey Smith G. Schneider M. Minder C. (1997). Bias in meta-analysis detected by a simple, graphical test. BMJ 315, 629–634. doi: 10.1136/bmj.315.7109.629 PMC2127453 9310563

[ref11] Fromm A. E. Trujillo-Llano C. Grittner U. Meinzer M. Flöel A. Antonenko D. (2025). Increased variability in response to transcranial direct current stimulation in healthy older compared to young adults: a systematic review and meta-analysis. Brain Stimul. 18, 1257–1265. doi: 10.1016/j.brs.2025.06.005, 40633904

[ref12] Grossman N. Bono D. Dedic N. Kodandaramaiah S. B. Rudenko A. Suk H.-J. . (2017). Noninvasive deep brain stimulation via temporally interfering electric fields. Cell 169, 1029–1041.e16. doi: 10.1016/j.cell.2017.05.024, 28575667 PMC5520675

[ref13] Hedges L. V. (1981). Distribution theory for glass's estimator of effect size and related estimators. J. Educ. Stat. 6, 107–128. doi: 10.3102/10769986006002107

[ref14] Higgins J. P. Thompson S. G. (2002). Quantifying heterogeneity in a meta-analysis. Stat. Med. 21, 1539–1558. doi: 10.1002/sim.1186 12111919

[ref15] Higgins J. P. Thompson S. G. Deeks J. J. Altman D. G. (2003). Measuring inconsistency in meta-analyses. BMJ 327, 557–560. doi: 10.1136/bmj.327.7414.557 PMC192859 12958120

[ref16] Hochstenbach J. van Spaendonck K. P. Cools A. R. Horstink M. W. Mulder T. (1998). Cognitive deficits following stroke in the basal ganglia. Clin. Rehabil. 12, 514–520. doi: 10.1191/026921598666870672, 9869255

[ref17] Homan S. Muscat W. Joanlanne A. Marousis N. Cecere G. Hofmann L. . (2021). Treatment effect variability in brain stimulation across psychiatric disorders: a meta-analysis of variance. Neurosci. Biobehav. Rev. 124, 54–62. doi: 10.1016/j.neubiorev.2020.11.033, 33482243

[ref18] Hsu T.-Y. Juan C.-H. Tseng P. (2016). Individual differences and state-dependent responses in transcranial direct current stimulation. Front. Hum. Neurosci. 10:643. doi: 10.3389/fnhum.2016.00643, 28066214 PMC5174116

[ref19] Hutcheon B. Yarom Y. (2000). Resonance, oscillation and the intrinsic frequency preferences of neurons. Trends Neurosci. 23, 216–222. doi: 10.1016/s0166-2236(00)01547-2 10782127

[ref20] Ivanov B. Toth J. E. Mandali A. Salvati R. Arvaneh M. (2025). Beyond the surface: a review of transcranial temporal interference stimulation for deep brain modulation. Front. Neurol. 16:1661049. doi: 10.3389/fneur.2025.1661049, 41059506 PMC12499357

[ref21] Jacobson L. Koslowsky M. Lavidor M. (2012). tDCS polarity effects in motor and cognitive domains: a meta-analytical review. Exp. Brain Res. 216, 1–10. doi: 10.1007/s00221-011-2891-9, 21989847

[ref22] Jaroudi W. Garami J. Garrido S. Hornberger M. Keri S. Moustafa A. A. (2017). Factors underlying cognitive decline in old age and Alzheimer’s disease: the role of the hippocampus. Rev. Neurosci. 28, 705–714. doi: 10.1515/revneuro-2016-0086, 28422707

[ref23] Lasogga L. Gramegna C. Müller D. Habel U. Mehler D. M. Gur R. C. . (2024). Meta-analysis of variance in tDCS effects on response inhibition. Sci. Rep. 14:19197. doi: 10.1038/s41598-024-70065-7, 39160262 PMC11333595

[ref24] Lee H. Choi B. J. Kang N. (2024). Non-invasive brain stimulation enhances motor and cognitive performances during dual tasks in patients with Parkinson’s disease: a systematic review and meta-analysis. J. Neuroeng. Rehabil. 21:205. doi: 10.1186/s12984-024-01505-8, 39581969 PMC11587594

[ref25] Lee J. H. Lee T. L. Kang N. (2021). Transcranial direct current stimulation decreased cognition-related reaction time in older adults: a systematic review and meta-analysis. Ageing Res. Rev. 70:101377. doi: 10.1016/j.arr.2021.101377, 34089900

[ref26] Lee T. L. Lee H. Kang N. (2023). A meta-analysis showing improved cognitive performance in healthy young adults with transcranial alternating current stimulation. NPJ Sci. Learn. 8:1. doi: 10.1038/s41539-022-00152-9, 36593247 PMC9807644

[ref27] Li T. Yang C. Xu Y. Du Y. Shen X. Zhou J. . (2026). Transcranial temporal interference stimulation of the globus pallidus internus ameliorates gait variability in early-to mid-stage Parkinson’s disease: a pilot study. Brain Stimul. 19:103007. doi: 10.1016/j.brs.2025.103007, 41419092

[ref28] López-Alonso V. Cheeran B. Río-Rodríguez D. Fernández-del-Olmo M. (2014). Inter-individual variability in response to non-invasive brain stimulation paradigms. Brain Stimul. 7, 372–380. doi: 10.1016/j.brs.2014.02.004, 24630849

[ref29] Ma R. Xia X. Zhang W. Lu Z. Wu Q. Cui J. . (2021). High gamma and beta temporal interference stimulation in the human motor cortex improves motor functions. Front. Neurosci. 15:800436. doi: 10.3389/fnins.2021.800436, 35046771 PMC8761631

[ref30] Mansourinezhad P. Mestrom R. M. Klooster D. C. Sprengers M. Boon P. A. Paulides M. M. (2025). Systematic review of experimental studies in humans on transcranial temporal interference stimulation. J. Neural Eng. 22:051001. doi: 10.1088/1741-2552/ae0524, 40925398

[ref31] McCutcheon R. A. Pillinger T. Mizuno Y. Montgomery A. Pandian H. Vano L. . (2021). The efficacy and heterogeneity of antipsychotic response in schizophrenia: a meta-analysis. Mol. Psychiatry 26, 1310–1320. doi: 10.1038/s41380-019-0502-5, 31471576 PMC7610422

[ref32] Minarik T. Berger B. Althaus L. Bader V. Biebl B. Brotzeller F. . (2016). The importance of sample size for reproducibility of tDCS effects. Front. Hum. Neurosci. 10:453. doi: 10.3389/fnhum.2016.00453, 27679568 PMC5020062

[ref33] Nakagawa S. Poulin R. Mengersen K. Reinhold K. Engqvist L. Lagisz M. . (2015). Meta-analysis of variation: ecological and evolutionary applications and beyond. Methods Ecol. Evol. 6, 143–152. doi: 10.1111/2041-210x.12309

[ref34] Nitsche M. A. Bikson M. Bestmann S. (2015). On the use of meta-analysis in neuromodulatory non-invasive brain stimulation. Brain Stimul. 8, 666–667. doi: 10.1016/j.brs.2015.03.008, 25937023

[ref35] Obeso J. A. Rodríguez-Oroz M. C. Benitez-Temino B. Blesa F. J. Guridi J. Marin C. . (2008). Functional organization of the basal ganglia: therapeutic implications for Parkinson's disease. Mov. Disord. 23, S548–S559. doi: 10.1002/mds.22062, 18781672

[ref36] Page M. J. McKenzie J. E. Bossuyt P. M. Boutron I. Hoffmann T. C. Mulrow C. D. . (2021). The PRISMA 2020 statement: an updated guideline for reporting systematic reviews. BMJ 372:n71. doi: 10.1136/bmj.n71, 33782057 PMC8005924

[ref37] Patel R. Ashcroft J. Patel A. Ashrafian H. Woods A. J. Singh H. . (2019). The impact of transcranial direct current stimulation on upper-limb motor performance in healthy adults: a systematic review and meta-analysis. Front. Neurosci. 13:1213. doi: 10.3389/fnins.2019.01213, 31803003 PMC6873898

[ref38] Piao Y. Ma R. Weng Y. Fan C. Xia X. Zhang W. . (2022). Safety evaluation of employing temporal interference transcranial alternating current stimulation in human studies. Brain Sci. 12:1194. doi: 10.3390/brainsci12091194, 36138930 PMC9496688

[ref39] Pick J. L. Nakagawa S. Noble D. W. (2019). Reproducible, flexible and high-throughput data extraction from primary literature: the metaDigitise r package. Methods Ecol. Evol. 10, 426–431. doi: 10.1111/2041-210x.13118

[ref40] Rampersad S. Roig-Solvas B. Yarossi M. Kulkarni P. P. Santarnecchi E. Dorval A. D. . (2019). Prospects for transcranial temporal interference stimulation in humans: a computational study. NeuroImage 202:116124. doi: 10.1016/j.neuroimage.2019.116124, 31473351 PMC6819277

[ref41] Senior A. M. Viechtbauer W. Nakagawa S. (2020). Revisiting and expanding the meta-analysis of variation: the log coefficient of variation ratio. Res. Synth. Methods 11, 553–567. doi: 10.1002/jrsm.1423, 32431099

[ref42] Sun J. H. Wu H. X. Chen Y. S. Zhang M. Qian Z. Y. Lü J. J. . (2025). Temporal interference stimulation modulates verbal working memory: an EEG study on hemispheric lateralization and neural dynamics. Cereb. Cortex 35:11. doi: 10.1093/cercor/bhaf102, 40751659

[ref43] Tang D. Qin L. Hu L. Jian Y. Gao S. Huang Z. . (2026). Personalized high-intensity temporal interference stimulation decouples cerebellar networks to enhance implicit learning. J. Neuroeng. Rehabil. 23:54. doi: 10.1186/s12984-025-01865-9, 41484914 PMC12866286

[ref44] Thiele C. Rufener K. S. Repplinger S. Zaehle T. Ruhnau P. (2024). Transcranial temporal interference stimulation (tTIS) influences event-related alpha activity during mental rotation. Psychophysiology 61:e14651. doi: 10.1111/psyp.14651, 38997805

[ref45] Vassiliadis P. Beanato E. Wessel M. J. Hummel F. C. (2026). Temporal interference stimulation for deep brain neuromodulation in humans. Nat. Biomed. Eng. 10, 1279–1294. doi: 10.1038/s41551-026-01665-z 42157014

[ref46] Vassiliadis P. Stiennon E. Windel F. Wessel M. J. Beanato E. Hummel F. C. (2024). Safety, tolerability and blinding efficiency of non-invasive deep transcranial temporal interference stimulation: first experience from more than 250 sessions. J. Neural Eng. 21:024001. doi: 10.1088/1741-2552/ad2d32, 38408385

[ref47] Vergallito A. Feroldi S. Pisoni A. Romero Lauro L. J. (2022). Inter-individual variability in tDCS effects: a narrative review on the contribution of stable, variable, and contextual factors. Brain Sci. 12:522. doi: 10.3390/brainsci12050522, 35624908 PMC9139102

[ref48] Viechtbauer W. (2010). Conducting meta-analyses in R with the metafor package. J. Stat. Softw. 36, 1–48. doi: 10.18637/jss.v036.i03

[ref49] Viechtbauer W. Cheung M. W. L. (2010). Outlier and influence diagnostics for meta-analysis. Res. Synth. Methods 1, 112–125. doi: 10.1002/jrsm.11, 26061377

[ref50] Violante I. R. Alania K. Cassarà A. M. Neufeld E. Acerbo E. Carron R. . (2023). Non-invasive temporal interference electrical stimulation of the human hippocampus. Nat. Neurosci. 26, 1994–2004. doi: 10.1038/s41593-023-01456-8, 37857775 PMC10620081

[ref51] Wagner T. Fregni F. Fecteau S. Grodzinsky A. Zahn M. Pascual-Leone A. (2007). Transcranial direct current stimulation: a computer-based human model study. NeuroImage 35, 1113–1124. doi: 10.1016/j.neuroimage.2007.01.027 17337213

[ref52] Wang C. Chen H. Liu M. Lu W. Hao Z. Wang B. (2026). Efficacy of non-invasive neuromodulation technologies in improving cognitive function and activities of daily living in patients with Alzheimer’s disease, Parkinson’s disease, and stroke: a systematic review and network meta-analysis. J. Neuroeng. Rehabil. 23:46. doi: 10.1186/s12984-025-01842-2, 41549257 PMC12853689

[ref53] Wang D. Du Z. Wen X. Li Q. Liu Y. Tang J. . (2025). Hippocampal transcranial temporal interference stimulation reduced craving in methamphetamine use disorder. Addict. Behav. 174:108581. doi: 10.1016/j.addbeh.2025.108581, 41389502

[ref54] Wang Y. Zeng G. Q. Wang M. Zhang M. Chang C. Liu Q. . (2024). The safety and efficacy of applying a high-current temporal interference electrical stimulation in humans. Front. Hum. Neurosci. 18:1484593. doi: 10.3389/fnhum.2024.1484593, 39677408 PMC11638170

[ref55] Wessel M. J. Beanato E. Popa T. Windel F. Vassiliadis P. Menoud P. . (2023). Noninvasive theta-burst stimulation of the human striatum enhances striatal activity and motor skill learning. Nat. Neurosci. 26, 2005–2016. doi: 10.1038/s41593-023-01457-7, 37857774 PMC10620076

[ref56] Winker M. Hoffmann S. Laborde S. Javelle F. (2024). The acute effects of motor cortex transcranial direct current stimulation on athletic performance in healthy adults: a systematic review and meta-analysis. Eur. J. Neurosci. 60, 5086–5110. doi: 10.1111/ejn.16488, 39120435

[ref57] Yang C. Xu Y. Feng X. Wang B. Du Y. Wang K. . (2025). Transcranial temporal interference stimulation of the right globus pallidus in Parkinson's disease. Mov. Disord. 40, 1061–1069. doi: 10.1002/mds.29967, 39133053 PMC12160976

[ref58] Zhao H. Zhao B. He J. Liu M. (2025). Distinctive roles of left and right entorhinal cortex in path integration via a non-invasive stimulation study. Nat. Commun. 16:10214. doi: 10.1038/s41467-025-64986-8, 41266335 PMC12635200

[ref59] Zheng S. Fu T. Yan J. Zhu C. Li L. Qian Z. . (2024). Repetitive temporal interference stimulation improves jump performance but not the postural stability in young healthy males: a randomized controlled trial. J. Neuroeng. Rehabil. 21:38. doi: 10.1186/s12984-024-01336-7, 38509563 PMC10953232

[ref60] Zheng S. Zhang Y. Huang K. Zhuang J. Lü J. Liu Y. (2025). Temporal interference stimulation boosts working memory performance in the Frontoparietal network. Hum. Brain Mapp. 46:e70160. doi: 10.1002/hbm.70160, 39936622 PMC11815565

