# Supplementary material for: Modulation of cognitive and motor functions by temporal interference stimulation: a systematic review and meta-analysis of efficacy and variability
Source: Front Psychol. 2026 Sep 11;17:1932755. doi: 10.3389/fpsyg.2026.1932755 (PMC13612444; doi:10.3389/fpsyg.2026.1932755)
Supplement: Supplementary file 1 [file Supplementary_Material_file_1.docx]

***Supplementary Material***

**Supplementary Tables**

Table S1. Leave-one-out sensitivity analysis for the SMD meta-analysis	2

Table S2. Leave-one-out sensitivity analysis for the lnCVR meta-analysis	3

**Supplementary Figures**

Fig. S1. Funnel plot of mean performance effects	4

Fig. S2. Funnel plot of response variability effects	5

**Table S1. Leave-one-out sensitivity analysis for the SMD meta-analysis.**

| Omitted Study | Pooled SMD | 95% CI | I² (%) | |
| --- | --- | --- | --- | --- |
| Beanato et al., 2024 | 0.31 | [0.165, 0.461] | 3.5 |  |
| Chen et al., 2026 | 0.31 | [0.152, 0.462] | 8.5 |  |
| Li et al., 2026 | 0.31 | [0.170, 0.453] | 0.0 |  |
| Ma et al., 2021a | 0.27 | [0.127, 0.413] | 0.0 |  |
| Ma et al., 2021b | 0.27 | [0.128, 0.418] | 0.0 |  |
| Sun et al., 2025 | 0.32 | [0.175, 0.461] | 0.0 |  |
| Tang et al., 2026 | 0.31 | [0.169, 0.458] | 1.0 |  |
| Thiele et al., 2024 | 0.30 | [0.160, 0.447] | 1.7 |  |
| Violante et al., 2023a | 0.31 | [0.160, 0.452] | 3.2 |  |
| Violante et al., 2023b | 0.31 | [0.168, 0.456] | 0.7 |  |
| Wang et al., 2024 | 0.32 | [0.179, 0.469] | 0.0 |  |
| Wang et al., 2025 | 0.29 | [0.142, 0.428] | 0.0 |  |
| Wessel et al., 2023a | 0.31 | [0.170, 0.454] | 0.0 |  |
| Wessel et al., 2023b | 0.31 | [0.169, 0.453] | 0.0 |  |
| Wessel et al., 2023c | 0.31 | [0.172, 0.456] | 0.0 |  |
| Yang et al., 2025 | 0.27 | [0.132, 0.414] | 0.0 |  |
| Zhao et al., 2025 | 0.28 | [0.134, 0.420] | 0.0 |  |
| Zheng et al., 2024 | 0.30 | [0.153, 0.444] | 3.1 |  |
| Zheng et al., 2025 | 0.29 | [0.146, 0.438] | 2.4 |  |

Each row presents the pooled standardized mean difference (SMD; Hedges’ g) obtained after sequentially omitting one study from the meta-analysis. The table reports the resulting pooled effect size, corresponding 95% confidence interval, and heterogeneity (I²) for each iteration. Abbreviations: CI, confidence interval; SMD, standardized mean difference.

**Table S2. Leave-one-out sensitivity analysis of the lnCVR meta-analysis.**

| Omitted Study | Pooled lnCVR | 95% CI | I² (%) |
| --- | --- | --- | --- |
| Beanato et al., 2024 | -0.13 | [-0.269, 0.012] | 0.0 |
| Li et al., 2026 | -0.13 | [-0.264, 0.010] | 0.0 |
| Sun et al., 2025 | -0.12 | [-0.261, 0.014] | 0.0 |
| Thiele et al., 2024 | -0.13 | [-0.267, 0.010] | 0.0 |
| Violante et al., 2023a | -0.12 | [-0.257, 0.023] | 0.0 |
| Violante et al., 2023b | -0.12 | [-0.262, 0.017] | 0.0 |
| Wang et al., 2024 | -0.11 | [-0.249, 0.025] | 0.0 |
| Wang et al., 2025 | -0.13 | [-0.271, 0.009] | 0.0 |
| Wessel et al., 2023a | -0.10 | [-0.242, 0.034] | 0.0 |
| Wessel et al., 2023b | -0.11 | [-0.249, 0.027] | 0.0 |
| Wessel et al., 2023c | -0.11 | [-0.246, 0.030] | 0.0 |
| Yang et al., 2025 | -0.11 | [-0.252, 0.024] | 0.0 |
| Zhao et al., 2025 | -0.11 | [-0.253, 0.025] | 0.0 |
| Zheng et al., 2024 | -0.07 | [-0.209, 0.072] | 0.0 |
| Zheng et al., 2025 | -0.12 | [-0.256, 0.027] | 0.0 |

Each row presents the pooled log coefficient of variation ratio (lnCVR) obtained after sequentially omitting one study from the meta-analysis. The table reports the resulting pooled effect size, corresponding 95% confidence interval, and heterogeneity (I²) for each iteration. Abbreviations: CI, confidence interval; lnCVR, log coefficient of variation ratio.

**Fig. S1. Funnel plot of mean performance effects**. Funnel plot illustrating the distribution of standardized mean differences (SMD; Hedges’ g) comparing temporal interference stimulation (TIS) with the control condition across 19 outcomes. Each point represents an individual study outcome. The vertical dashed line indicates the pooled effect estimate; the triangular region represents the pseudo-95% confidence limits.

**Fig. S2. Funnel plot of response variability effects**. Funnel plot illustrating the distribution of log coefficient of variation ratios (lnCVR) comparing temporal interference stimulation (TIS) with the control condition across 15 outcomes. Each point represents an individual study outcome. The vertical dashed line indicates the pooled effect estimate, and the triangular region represents the pseudo-95% confidence limits. Open circles represent imputed studies estimated by the trim-and-fill method.
